# Bacterial denitrification drives elevated N_2_O emissions in arid southern California drylands

**DOI:** 10.1126/sciadv.adj1989

**Published:** 2023-12-06

**Authors:** Alexander H. Krichels, G. Darrel Jenerette, Hannah Shulman, Stephanie Piper, Aral C. Greene, Holly M. Andrews, Jon Botthoff, James O. Sickman, Emma L. Aronson, Peter M. Homyak

**Affiliations:** ^1^ Environmental Sciences, University of California, Riverside, CA, USA.; ^2^ Center for Conservation Biology, University of California, Riverside, CA, USA.; ^3^ USDA Rocky Mountain Research Station, Albuquerque, NM, USA.; ^4^ Botany and Plant Sciences, University of California, Riverside, CA, USA.; ^5^ Ecology and Evolutionary Biology, University of Tennessee, Knoxville, TN, USA.; ^6^ Microbiology and Plant Pathology, University of California, Riverside, CA, USA.; ^7^ Houston Advanced Research Center, The Woodlands, TX, USA.; ^8^ Evolution, Ecology, and Organismal Biology, University of California, Riverside, CA, USA.; ^9^ Geography, Development and Environment, University of Arizona, Tucson, AZ, USA.

## Abstract

Soils are the largest source of atmospheric nitrous oxide (N_2_O), a powerful greenhouse gas. Dry soils rarely harbor anoxic conditions to favor denitrification, the predominant N_2_O-producing process, yet, among the largest N_2_O emissions have been measured after wetting summer-dry desert soils, raising the question: Can denitrifiers endure extreme drought and produce N_2_O immediately after rainfall? Using isotopic and molecular approaches in a California desert, we found that denitrifiers produced N_2_O within 15 minutes of wetting dry soils (site preference = 12.8 ± 3.92 per mil, δ^15^N^bulk^ = 18.6 ± 11.1 per mil). Consistent with this finding, we detected nitrate-reducing transcripts in dry soils and found that inhibiting microbial activity decreased N_2_O emissions by 59%. Our results suggest that despite extreme environmental conditions—months without precipitation, soil temperatures of ≥40°C, and gravimetric soil water content of <1%—bacterial denitrifiers can account for most of the N_2_O emitted when dry soils are wetted.

## Teaser

Despite dry and hot summers, denitrifiers endure through months in deserts and produce large N2O emissions after rainfall.

## INTRODUCTION

Nitrous oxide (N_2_O) is increasing in Earth’s atmosphere, catalyzing the destruction of stratospheric ozone and warming the planet ~273 times more effectively than carbon dioxide on a per molecule basis (*1*–*3*). Over a quarter of atmospheric N_2_O originates from natural soils (*2*), which harbor microbial communities that anaerobically produce N_2_O when wet conditions limit oxygen diffusion. Ecosystems characterized by dry soils do not often generate the wet conditions required to limit oxygen diffusion and are, therefore, not considered major sources of N_2_O (*4*–*6*). However, unexpectedly, among the highest instantaneous N_2_O emission rates (i.e., emission pulses) have been recorded within minutes of adding water to dry desert soils experiencing extreme desiccation and summer heat (*7*, *8*). Thus, understanding how dry conditions affect the processes that produce N_2_O can help forecast atmospheric N_2_O concentrations as drought becomes more common across terrestrial ecosystems (*9*).

The sequential anaerobic reduction of nitrate (NO_3_^−^) to N_2_O by denitrification and the aerobic oxidation of ammonia (NH_3_) to NO_3_^−^ by nitrification are two of the predominant processes producing N_2_O in soils (*4*, *10*). In drylands, where infrequent rainfall may rarely develop the anoxic soil environments required for denitrification, biogeochemical theory would predict that oxygen reduction by nitrifiers is thermodynamically favored over NO_3_^−^ reduction by denitrifiers (*4*–*6*). However, in deserts, extreme heat and aridity may limit the survival and activity of microorganisms (*10*–*14*), suggesting that the rapid N_2_O emission pulses detected within minutes of wetting soils may not be exclusively biological. N_2_O can be produced via chemodenitrification, an abiotic process coupling the reaction of metals with nitrite (NO_2_^−^) or hydroxylamine (NH_2_OH) (*15*–*18*). However, the N_2_O emission pulses measured after wetting dry soils are at least partly derived from NO_3_^−^ (*7*, *8*, *19*, *20*) and not exclusively from NO_2_^−^ or NH_2_OH as chemodenitrification would predict. Given that (i) the abiotic reduction of NO_3_^−^ has only been reported in heavily manipulated laboratory mesocosms (*21*–*24*) and (ii) extremely dry and hot conditions may limit the survival and activity of microorganisms, the mechanisms producing N_2_O emission pulses after wetting dry soils experiencing extreme desiccation and summer heat remain unclear.

Determining which processes reduce NO_3_^−^ to N_2_O under extreme desiccation and summer heat may be possible by combining isotopic and molecular approaches. N_2_O is an asymmetric linear molecule, where the difference in isotopic composition between the two N atoms in N_2_O—the “site preference” (or SP)—varies as a function of the relative contribution of nitrification, nitrifier denitrification, bacterial denitrification, fungal denitrification, chemodenitrification, and N_2_O reduction to N_2_ (*25*–*29*). In addition to SP, bulk ^15^N (δ^15^N^bulk^) and ^18^O values can also help identify the many processes that produce and consume N_2_O (*25*, *28*). However, because some processes produce overlapping effects in N_2_O isotope space (*25*, *28*, *29*), isotope tracers can help resolve whether NO_3_^−^ or NH_4_^+^ are converted to N_2_O, and quantitative polymerase chain reaction (qPCR) can be used to assess the abundance of denitrification genes in soils. Here, we combined isotopic and molecular analyses to ask: Following extended hot and dry periods known to limit anoxic conditions and constrain denitrification, can denitrifiers rapidly reduce NO_3_^−^ to N_2_O?

We answered this question by studying four arid sites (labeled A to D) in southern California, USA, with site A being the wettest [299-mm mean annual precipitation (MAP)] and sites B to D becoming increasingly drier (down to 101-mm MAP; Table 1). We hypothesized that despite the hot and dry conditions known to hinder microbial denitrification, denitrifiers can endure through extreme desiccation and heat (soil temperature often exceeding 40°C with gravimetric soil water content of <1%) and are key to producing the unexpectedly large N_2_O emissions when dry desert soils are wetted. We found that N_2_O produced from these desert soils had isotopic values consistent with bacterial denitrification [SP = 12.8 ± 3.92 per mil (‰), δ^15^N^bulk^ = 18.6 ± 11.1‰], that desert soils maintained NO_3_^−^-reducing genes and transcripts under extreme desiccation and heat before our wetting experiments, and that slowing microbial activity with chloroform decreased the reduction of NO_3_^−^ to N_2_O by 59%. Together, these results show that bacterial denitrification can reduce NO_3_^−^ to N_2_O within minutes of wetting dry soils and contribute to rapid N_2_O emission pulses observed across many dry lands.

**Table 1.** Site characteristics at our four studied sites.

	Site A	Site B	Site C	Site D
Latitude	33.9221	33.8961	33.9440	33.9041
Longitude	−116.7577	−116.6868	−116.3949	−115.7233
Total C (%)	1.69 ± 0.73	0.99 ± 0.66	0.83 ± 0.63	0.54 ± 0.29
Total N (%)	0.15 ± 0.060	0.085 ± 0.056	0.066 ± 0.035	0.050 ± 0.024
pH	6.80 ± 0.09	6.84 ± 0.39	7.44 ± 0.16	8.03 ± 0.30
Soil δ^15^N (‰)	4.23 ± 0.57	5.38 ± 0.30	4.28 ± 0.91	7.20 ± 1.31
NO_3_^−^ (μg of N g dry soil^−1^)	5.48 ± 3.46	7.08 ± 3.95	2.75 ± 1.11	2.76 ± 1.81
NH_4_^+^ (μg of N g dry soil^−1^)	8.92 ± 5.67	8.37 ± 3.39	7.86 ± 9.34	1.62 ± 1.09
NO_2_^−^ (μg of N g dry soil^−1^)	0.48 ± 0.26	NA	0.15 ± 0.07	0.062 ± 0.088
Modeled N deposition (kg of N ha^−1^)*	9.3	8.2	4.5	3.0
Ambient NO*_x_* concentration (ppb)†	9.9	4.2	2.2	1.5
MAP (mm)‡	299	246	145	101

*Modeled atmospheric N deposition estimates were obtained from Schwede and Lear and the National Atmospheric Deposition Program (*70*).

†Measured atmospheric NO*_x_* concentrations reported in Krichels *et al.* (*71*).

‡MAP was obtained from Daly *et al.* (*72*).

## RESULTS

### Field N_2_O emissions and isotope values

**Fig. 1.**
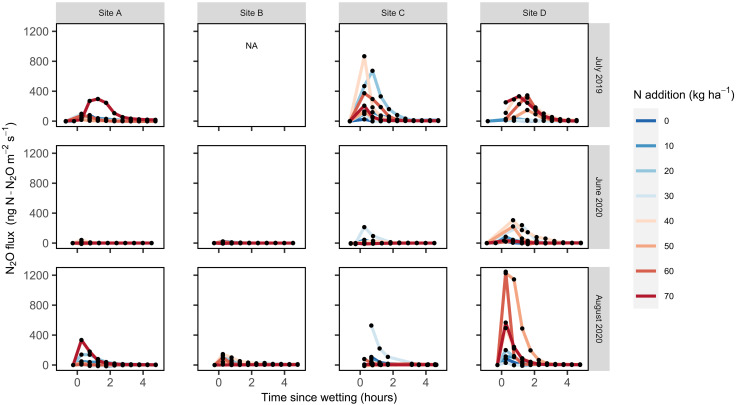
Field N_2_O emissions (in nanograms of N-N_2_O per square meter per second) over 5 hours after wetting summer-dry soils with ^15^N-nitrate solutions. Each black dot represents flux measurements over a 2-min period for each of the eight automated chambers under N treatment (line colors correspond to levels of N enrichment; in kilograms per hectare). NA, data not available. The units and scale on all *x* and *y* axes are the same on each panel.

Wetting dry soils with ^15^N-NO_3_^−^ tracer solution (to simulate a ~7-mm rain event) at concentrations ranging from 0 to 70 kg of N ha^−1^ stimulated N_2_O emissions. In July 2019 and August 2020, N_2_O was stimulated in all four sites, whereas in June 2020, N_2_O was stimulated in sites C and D (Tables 1 and 2), with emissions usually peaking within 1 hour of wetting and returning to baseline within 4 hours (Fig. 1). The magnitude of the N_2_O peak (i.e., the highest N_2_O emission rate measured after wetting dry soil) in response to adding the NO_3_^−^ tracer solution varied across sites, averaging 414 ± 405 ng of N-N_2_O m^−2^ s^−1^ in site D in August 2020, but only 83.5 ± 125 ng of N-N_2_O m^−2^ s^−1^ in site A during the same sampling campaign (August 2020; averages include N addition amounts ranging from 0 to 70 kg of N ha^−1^).

**Table 2.** Summary of field and laboratory experiments conducted in our study.

	Year	Sites	Description
Field N_2_O emissions	July 2019	A, C, and D	Measured field N_2_O emissions after wetting soils with ^15^N-NO_3_^−^ or ^15^N-NH_4_^+^ tracer solutions ranging from 0 to 70 kg of N ha^−1^.
	June 2020	A, B, C, and D	Measured field N_2_O emissions after wetting soils with either NO_3_^−^ or NH_4_^+^ solutions (sites A to C) or ^15^N-NO_3_^−^ or ^15^N-NH_4_^+^ tracer solutions (site D) ranging from 0 to 70 kg of N ha^−1^.
	August 2020	A, B, C, and D	Measured field N_2_O emissions after wetting soils with NO_3_^−^ or NH_4_^+^ solutions ranging from 0 to 70 kg of ha^−1^.
			
qPCR	July 2019	A and C	Measured the abundance of NO_3_^−^-reducing genes and transcripts from dry soils collected from field sites immediately before the wetting experiments.
	June 2020	D	
			
Chloroform inhibition	2021	D	Measured N_2_O emissions and ^15^N-N_2_O from dry soils incubated in microcosms in the laboratory. Soils were wetted with ^15^N-NO_3_^−^ tracer solution after being exposed to either chloroform or ambient laboratory air (control) before wetting.
			
Natural abundance N_2_O isotopes	2021	D	Measured the natural abundance isotopic composition of N_2_O (SP, δ^15^N^bulk^, and δ^18^O) after wetting dry soils in laboratory incubations.

**Fig. 2.**
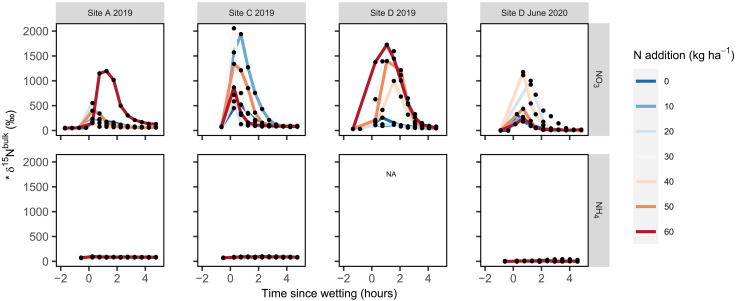
Isotopic composition (*δ^15^N) of field N_2_O emissions over 5 hours after wetting summer-dry soils with either ^15^N-NO_3_^−^ or ^15^N-NH_4_^+^ solutions. Each black dot represents the average isotopic composition of N_2_O using the last 10 s of a 2-min measurement from each chamber. Line colors correspond to levels of N enrichment (in kilograms per hectare). We use *δ^15^N to indicate uncertainty in isotope values given the open system chamber methodology used (see Materials and Methods). The units and scale on all *x* and *y* axes are the same on each panel.

While adding ^15^N-NO_3_^−^ tracer solutions stimulated N_2_O emissions, peak N_2_O emissions were only positively correlated to the amount of NO_3_^−^ added in site D in July 2019 (*P* = 0.008, *R*^2^ = 0.067; Fig. 1 and fig. S3) and August 2020 (*P* = 0.036, *R*^2^ = 0.47; Fig. 1 and fig. S3). Still, ^15^N-NO_3_^−^ was reduced to form N_2_O within 15 min of being added at all sites that received the label (sites A, C, and D in 2019 and site D in 2020; Table 2), producing peak δ^15^N^bulk^ values (defined as the highest δ^15^N^bulk^ measurement from a given chamber over the 24 hours after wetting) that averaged 778 ± 591‰ and often surpassed 1000‰ (Fig. 2). In contrast to adding NO_3_^−^, peak N_2_O emissions were not correlated to the amount of NH_4_^+^ added at any of the sites (*P* > 0.05; table S3 and fig. S4) with relatively small amounts of the ^15^N-NH_4_^+^ label transferred to N_2_O; peak δ^15^N^bulk^ values averaged 68 ± 39‰ and never exceeded 103‰ (Fig. 2).

## qPCR: Abundance of NO_3_^−^-reducing microbes

**Fig. 3.**
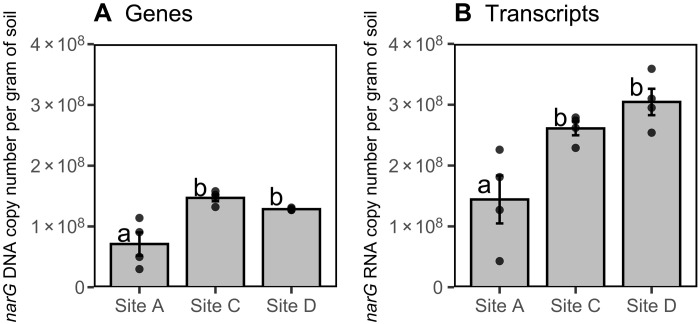
Abundance of nitrate reducing genes and transcripts in dry soils. Copy number of *narG* gDNA (**A**) and cDNA (**B**) from dry soil. Bars represent mean copy number (*n* = 4), error bars represent SEM, and dots represent individual measurements. Lower case letters represent significant differences in the means (*P* < 0.05) using Tukey corrected multiple comparisons.

The abundance of NO_3_^−^-reducing microorganisms (based on *narG* genes amplified from DNA that encode for the production of NO_3_^−^-reducing enzymes) before wetting dry soils differed among sites (*F*_2,9_ = 11.9, *P* = 0.003; Fig. 3A) and was highest at the sites with lowest annual precipitation and highest soil pH. Site A had significantly fewer *narG* gene copies (7.11 × 10^7^ ± 3.82 × 10^7^ copies g^−1^ of soil) than site C (*P* = 0.003; 1.47 × 10^8^ ± 1.11 × 10^7^ copies g^−1^ of soil) or site D (*P* = 0.016; 1.29 × 10^8^ ± 1.91 × 10^6^ copies g^−1^ of soil); site B was not measured because of limited resources. Similarly, the activity of NO_3_^−^-reducing microorganisms (based on *narG* transcripts amplified from mRNA) differed in dry soils among sites (*F*_2,9_ = 9.60, *P* = 0.006; Fig. 3B), with site A having significantly fewer copies (1.44 × 10^8^ ± 7.87 × 10^7^ copies g^−1^ of soil) than site C (*P* = 0.03; 2.61 × 10^8^ ± 2.25 × 10^7^ copies g^−1^ of soil) or site D (*P* = 0.006; 3.05 × 10^8^ ± 4.34 × 10^7^ copies g^−1^ of soil). In contrast to *narG* genes, we detected fewer than 2.0 × 10^4^ *napA* gene copies g^−1^ of soil, which also encode for NO_3_^−^-reducing enzymes. *napA* gene copy number did not differ by site (*F*_2,9_ = 0.35, *P* = 0.71), and we did not detect *napA* transcripts in our samples.

### N_2_O emissions from chloroform-fumigated soils labeled with ^15^N-NO_3_− tracer

**Fig. 4.**
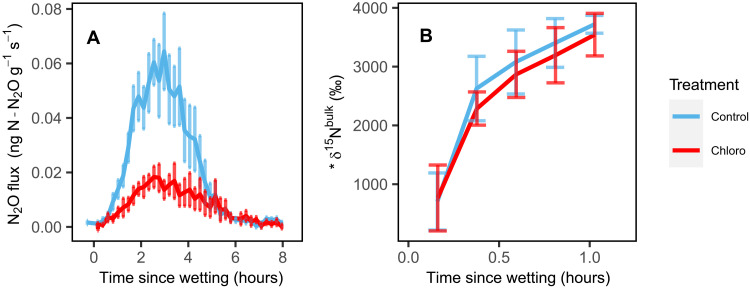
N_2_O emissions and N_2_O isotopic composition from chloroform incubated soils in the lab. Soil N_2_O emissions (**A**) and N_2_O isotopic composition (δ^15^N^bulk^) (**B**) from site D soils after wetting with a ^15^N-NO_3_^−^ solution in laboratory closed-system incubations. Lines represent mean N_2_O emissions (*n* = 8), and error bars represent the SEM. Soils were incubated in a chloroform-enriched headspace (“Chloro”) or under ambient conditions (“Control”) for 10 days before wetting.

Soil N_2_O emissions decreased by 59% after fumigating soils from site D with CHCl_3_ in laboratory incubations (Fig. 4A; *t*_7,0.05_ = 4.14, *P* = 0.004). We only fumigated soils from site D since this site produced the most N_2_O in the field. Similar to the pulse dynamics we observed in the field, N_2_O emissions peaked within 4 hours of wetting with NO_3_^−^ solutions (soils were wet to 20% gravimetric soil moisture) for both fumigated and nonfumigated soils and returned to near zero within 6 hours (Fig. 4A). For both fumigated and nonfumigated soils, ^15^N-NO_3_^−^ was reduced to N_2_O within 25 min of wetting and produced similar δ^15^N^bulk^ values (Fig. 4B); δ^15^N^bulk^ reached 2614 ± 1553‰ within 25 min of wetting nonfumigated soils and 2287 ± 800‰ within 25 min of wetting CHCl_3_-fumigated soils (Fig. 4B).

### Natural abundance isotopocules of N_2_O

**Fig. 5.**
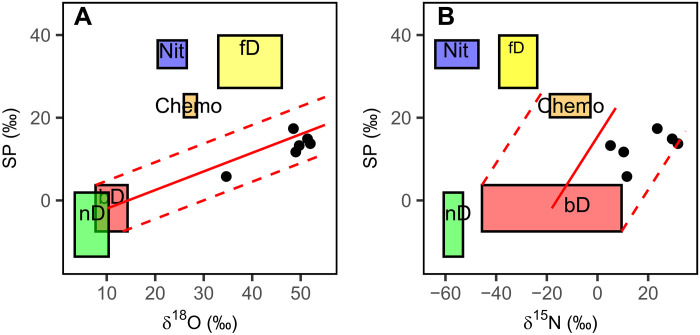
Natural abundance isotopic composition of N_2_O emitted during lab incubations. Dual natural abundance isotopic composition of SP and either δ^18^O (**A**) or δ^15^N^bulk^ (**B**) emitted after wetting summer-dry soils from site D in laboratory closed-system incubations. Black dots represent the isotopic composition of N_2_O collected over the course of 8 hours from six mesocosm incubations. Boxes represent literature-derived estimates of the isotopic composition of N_2_O produced from nitrification (nit), nitrifier denitrification (nD), bacterial denitrification (bD), fungal denitrification (fD), and chemodenitrification (chemo), which are reported on the basis of the assumption that all substrate isotope values (δ^18^O-H_2_O, δ^15^N-NO_3_^−^, and δ^15^N-NH_4_^+^) were 0‰. The expected δ^18^O-N_2_O values for bacterial denitrification, fungal denitrification, and nitrifier denitrification depend on δ^18^O-H_2_O; the δ^18^O-H_2_O of the deionized water used in this experiment (−9‰) was therefore added to the literature-derived δ^18^O-N_2_O values. Similarly, the δ^15^N^bulk^-N_2_O of bacterial denitrification and fungal denitrification depend on the isotope value of the substrate (δ^15^N-NO_3_^−^), while δ^15^N^bulk^-N_2_O from chemodenitrification depends on δ^15^N-NO_2_^−^; the combined δ^15^N-NO_3_^−^ and δ^15^N-NO_2_^−^ measured from site D (7.2‰) was therefore added to the literature-derived [δ^15^N^bulk^]N_2_O values. While δ^15^N^bulk^-N_2_O from nitrification and nitrifier denitrification depend on δ^15^N-NH_4_^+^, we did not measure δ^15^N-NH_4_^+^ in this study. For the purpose of this figure, δ^15^N-NH_4_^+^ was assumed to be 0‰. Even if δ^15^N-NH_4_^+^ was as enriched as [δ^15^N]NO_3_^−^ (7.2‰), this correction would not affect our interpretation of the data since the measured δ^15^N^bulk^-N_2_O was over 40‰ more enriched compared to expected values for nitrification or nitrifier denitrification. The solid red line shows the expected effect of N_2_O reduction to N_2_ on N_2_O isotope values, and the dashed lines show the range of possible effects of N_2_O reduction depending on the starting isotopic composition of N_2_O produced from bacterial denitrification.

After a 6-hour laboratory incubation at 20% gravimetric soil moisture, each mesocosm produced enough N_2_O [>0.6 parts per million (ppm)] to measure isotopocules from site D. We only measured natural abundance N_2_O isotopes from site D since this site produced the most N_2_O in the field. SP averaged 12.8 ± 3.9‰ (Fig. 5), outside of the ranges expected for N_2_O produced from bacterial denitrification (−7.5 to 3.7‰), fungal denitrification (27.2 to 39.9‰), and chemodenitrification (20.1 to 25.7‰; Fig. 5) (*25*). However, SP values matched the expected mixing ratio between the production of N_2_O via bacterial denitrification and the reduction of N_2_O to N_2_ (Fig. 5). The δ^15^N^bulk^ was relatively enriched in ^15^N (18.6 ± 11.1‰) along with δ^18^O being relatively enriched in ^18^O (47.5 ± 6.46‰).

## DISCUSSION

Using molecular and isotopic tools, we show that denitrifiers reduced NO_3_^−^ to N_2_O within minutes of wetting desert soils that had been dry for months under summer heat. Despite the low soil water content, denitrification genes and transcripts were detected in these dry soils before wetting, with postwetting N_2_O emissions in the laboratory producing isotopic values consistent with mixing between bacterial denitrification and N_2_O reduction to N_2_. Together, these results suggest that denitrification may be an often overlooked source of N_2_O emissions from ecosystems that may be perceived as too dry to support this process.

### Production of N_2_O via denitrification

Denitrification was rapidly up-regulated after wetting dry soils despite months of preceding dry and hot conditions known to hinder this biological process. Within 15 min of wetting summer-dry soils, we detected our ^15^N-NO_3_^−^ tracer in the emitted N_2_O, consistent with earlier work (*7*). Furthermore, we measured low SP values in laboratory incubations (12.8 ± 3.92‰; Fig. 5), consistent with values expected from mixing between bacterial denitrification and either chemodenitrification or N_2_O reduction to N_2_ (Fig. 5) (*25*, *28*). While these SP values do not rule out the production of N_2_O via nitrifier denitrification, this process reduces NO_2_^−^, not NO_3_^−^, and, thus, cannot explain incorporation of the ^15^N-NO_3_^−^ tracer into N_2_O that we observed in the field (Figs. 2 and 3B). However, because 12.8 ± 3.9‰ is outside of the range of SP values expected so far from bacterial denitrification (−7.5 to 3.7‰), other processes—e.g., chemodenitrification or N_2_O reduction to N_2_—likely contributed to the N_2_O emissions (Fig. 5). Nevertheless, the role of bacterial denitrifiers producing N_2_O is further supported by the relatively high δ^15^N^bulk^ (18.6 ± 11.1‰) measured in laboratory incubations, as bacterial denitrification may not discriminate against ^15^N-NO_3_^−^ to the same degree as other NO_3_^−^-reducing processes (*25*). We also observed ^15^N-NO_3_^−^ tracer in NO, a denitrification intermediate, measured within 15 min of wetting dry soil (fig. S5). Overall, our measurements suggest that denitrifiers were key to reducing NO_3_^−^ and producing N_2_O after wetting these dry desert soils.

In further support of rapid up-regulation of denitrification in dry soils, we detected *narG* genes and transcripts that encode for NO_3_^−^-reducing enzymes before wetting soils that had experienced months of summer desert heat (Fig. 3). This suggests that denitrifiers could have been active, even under dry conditions, and that they may be well equipped to up-regulate metabolism when soils wet up. Desert soils can support denitrifier communities (*11*, *30*–*32*), and wetting soils following experimental drought can stimulate denitrification (*19*, *20*). Because we did not detect *napA* transcripts, it is possible that *narG* denitrifiers are better suited to remain active in hot and dry environments (*33*–*35*) to take advantage of resources flushed during brief anoxic periods after wetting (*36*–*41*). While detecting *narG* transcripts does not conclusively demonstrate that biological processes were reducing NO_3_^−^—posttranscriptional factors (e.g., pH) can determine whether mRNA transcripts are translated into denitrification enzymes (*42*, *43*)—fumigating soils in the laboratory with CHCl_3_ decreased N_2_O emissions by 59% (Fig. 4), suggesting that microbial processes produced most of the N_2_O from these soils (*44*). Together, our ability to measure (i) *narG* transcripts in summer-dry desert soils, (ii) the incorporation of the ^15^N-NO_3_^−^ tracer in the N_2_O emitted from the field, (iii) the decrease in N_2_O emissions after lowering microbial activity with CHCl_3_, and (iv) the natural abundance isotopocules of N_2_O falling within the range of denitrification, suggests that denitrifiers can rapidly reduce NO_3_^−^ to N_2_O and have the capacity to endure through hot and dry summer characteristic of many ecosystems.

### Complete denitrification also contributes to N_2_O emissions

Bacterial denitrification produced N_2_O at our most arid site, but other abiotic or microbial processes must have also occurred for SP values to rise above the range expected for bacterial denitrification (−7.5 to 3.7‰; Fig. 5). Chemodenitrification could have reduced native soil NO_2_^−^ (*15*, *16*, *45*), elevating SP values to those observed in the laboratory incubation (Fig. 5). Indeed, there was enough native NO_2_^−^ in these soils for chemodenitrification to account for even the N_2_O pulses we observed in the field (Table 1). However, if chemodenitrification reduced native NO_2_^−^ to N_2_O, then we would expect δ^15^N^bulk^-N_2_O to decrease under chloroform fumigation due to (i) abiotic incorporation of unlabeled NO_2_^−^ into N_2_O and (ii) lower incorporation of ^15^N-NO_3_^−^ into N_2_O from denitrifiers, but this was not the case (Fig. 4B). While chemodenitrification may also be able to reduce NO_3_^−^ to explain the observed patterns, this has only been shown under heavily manipulated conditions (*22*), and it is not clear whether this process occurs under field conditions (*23*, *24*). Even if chemodenitrification did reduce NO_3_^−^, chemodenitrification has not been observed to produce N_2_O with δ^15^N^bulk^ above −10‰ and δ^18^O above 37.6‰ (in relation to the −9‰ δ^18^O of water used in this study), such that mixing between bacterial denitrification and chemodenitrification, alone, may not explain the relatively high δ^15^N^bulk^ (19 ± 11‰) and δ^18^O (48 ± 6‰) that we measured (Fig. 5) (*25*, *28*, *29*). Rather, the elevated natural abundance SP, δ^15^N^bulk^, and δ^18^O all correspond to the expected isotope effects of N_2_O reduction to N_2_ by denitrifiers (Fig. 5) (*25*, *46*), with the near equal δ^15^N^bulk^-N_2_O values between CHCl_3_ and control soils suggesting that some denitrifiers could have survived the CHCl_3_ fumigation (Fig. 4B). N_2_O reduction to N_2_ is an anaerobic process not often measured in dryland ecosystems (*30*, *47*), but many denitrifiers have both NO_3_^−^- and N_2_O-reducing genes (*4*, *48*), such that the same organisms that reduce NO_3_^−^ may also reduce N_2_O when wetting establishes anoxic conditions. Even if soils do not maintain anoxic microsites, a growing number of nondenitrifying organisms have been shown to reduce N_2_O under aerobic conditions, allowing for N_2_O reduction in aerated soils (*48*, *49*). Thus, while chemodenitrification may have occurred, bacterial denitrification and N_2_O reduction to N_2_ best explain the N_2_O isotope values we observed, indicating that anaerobic microbial processes play an important role in regulating N_2_O emissions after wetting dry soils.

### Denitrifier abundance may contribute to variation in N_2_O emissions among sites

We found that *narG* genes and transcripts were more abundant in the more arid sites (Fig. 3), potentially favoring high rates of NO_3_^−^ reduction to N_2_O upon wetting. It is possible that resource-limiting conditions (e.g., low C, N, and precipitation) in the more arid sites support extremophile bacteria that thrive during brief periods when wetting displaces soil O_2_ and flushes soil pores with C and NO_3_^−^ (*4*, *50*). In support of this argument, denitrifiers from the *Rubrobacter* genus were more abundant at the more arid sites during our study (*51*); these taxa can survive desiccation during high temperatures, tolerate ultraviolet radiation, and have *narG* and other denitrification genes (*51*–*53*). While we did not assess which microorganisms reduced NO_3_^−^ to N_2_O in our sites, our data suggest that determining the ecology of dryland NO_3_^−^-reducing microorganisms may help predict which drylands operate as N_2_O sources. For example, pulsed N_2_O emissions in the most arid site were of similar magnitude to those measured in a nearby desert site (*8*), perhaps suggesting that these sites could share similar microbiomes that could help predict function. Enhancing our ability to predict soil N emissions may be particularly important for drylands since N_2_O emissions may account for between 27 – 56% of atmospheric N inputs in some desert sites (*8*). Moreover, coarse estimates suggest that desert N_2_O emissions may be equivalent to ~11 to 20% of the annual N_2_O emissions per unit area from the U.S. corn belt (*8*), one of the largest emitters of N_2_O (*54*), suggesting that drylands can contribute to a substantial fraction of atmospheric N_2_O.

### Conclusion

By combining isotopic tools with molecular approaches in both the field and laboratory, we show that denitrification governed N_2_O emissions in these desert soils despite the extreme environmental conditions preceding experimental wetting events (i.e., months without precipitation, soil temperatures in excess of 40°C, and gravimetric soil water content of <1%; figs. S7 and S8). Our measurements suggest that even at environmental extremes, dry soils can still support denitrifiers and that microbial NO_3_^−^ reduction may be an important strategy for heterotrophic respiration in ecosystems experiencing extreme drought during key periods following rainfall. Accounting for pulses of denitrifier activity during drying-wetting events could help improve forecasts of atmospheric N_2_O concentrations from models that do not currently account for appreciable N_2_O emissions from dryland ecosystems.

## MATERIALS AND METHODS

### Sites descriptions

We studied four sites (labeled A to D) across an aridity gradient in southern California, with site A being the wettest (299-mm MAP) and sites B to D becoming increasingly drier (down to 101-mm MAP; Table 1). Because of the proximity of our sites to the city of Los Angeles, USA, the sites also fall along an atmospheric N deposition gradient, with the highest atmospheric N deposited in site A and sites B to D receiving successively less N (Table 1). Creosote shrubs (*Larrea tridentata*) were the dominant vegetation at all sites. Soils were derived from similar granitic parent material but varied in pH, texture, and taxonomy, with site A being the least alkaline and sites B to D becoming progressively more alkaline (Table 1 and table S1).

### Experimental design

We measured N_2_O emissions from soils underneath eight Creosote shrubs at each of the four sites in July 2019, June 2020, and August 2020. Because of rainfall interrupting our rewetting experiments in 2019, we were unable to measure emissions from site B, and we only measured emissions in response to adding NO_3_^−^ in site D. Emissions were measured in response to experimentally wetting soils underneath shrubs with 500 ml of deionized water with different amounts of dissolved NO_3_^−^ or NH_4_^+^. The volume of water added was chosen to simulate a 7-mm rain event, approximately the average size of a summer rain event at our sites (https://deepcanyon.ucnrs.org/weather-data/). In sites A, C, and D in July 2019 and in site D in June 2020, the N solutions were labeled with ^15^N-NO_3_^−^ or ^15^N-NH_4_^+^ enriched to 2 atomic percent (at %) of ^15^N (Table 2). We used ascorbic acid to ensure that the ^15^N-NO_3_^−^ solution was free of NO_2_^−^ contamination (*55*). For all other sampling campaigns (sites A to C in June 2020 and all sites in August 2020; Table 2), the N additions were not labeled with isotopically enriched ^15^N; these measurements were used to assess how N_2_O emissions changed in response to adding N. Measurements were made underneath shrub canopies to capture “islands of fertility” where soil nutrients are concentrated (*56*). The shrubs were separated from one another by at least 1 m and were all within a 10-m radius. Under each shrub canopy, two pairs of polyvinyl chloride collars (four collars total; 20 cm in diameter × 10 cm in height) were inserted 5 cm into the ground at least 48 hours before starting measurements. One pair of collars was wetted with either water or NO_3_^−^ solution, while the other pair was wetted with either water or NH_4_^+^ solution. Nitrogen concentrations in the wetting solutions corresponded to a range in annual N deposition rates observed in Southern California drylands, so that each shrub received a different amount of N: 0, 10, 20, 30, 40, 50, 60, or 70 kg of N ha^−1^ (*8*, *57*, *58*). While these N addition amounts increased soil inorganic N in the top 10 cm of the soil by between ~1.5 and ~4 times, lower N addition amounts (between 2 and 15 kg of N ha^−1^) have not stimulated N trace gas emissions in other desert soils (*7*, *8*, *59*). Thus, we used higher N amounts to maximize our ability to predict changes in N emissions from soil N availability. Collar pairs were installed at least 1 m apart to limit cross-contamination of isotope tracers between collars. N_2_O emissions were measured from the collars that were amended with N. The collars that were not amended with N were wetted with 500 ml of water at the same time that the tracer solution was added to the other collar within each pair. The collars that were wetted with water were used to measure soil temperature (Model 8150-203, LI-COR Biosciences) and moisture (Model 8150-205, LI-COR Biosciences) to avoid disturbing the soils in the collars that were used to measure N_2_O emissions. The NO_3_^−^ solution was added to soils at approximately 9:00 a.m. with N_2_O emissions measured from each shrub every 30 min over 24 hours, starting 15 min after wetting. This was then repeated with the NH_4_^+^ solution the following morning using the other pair of collars underneath each shrub.

We measured soil NO_3_^−^, NH_4_^+^, and NO_2_^−^ concentrations from dry soils before adding our water and N solutions. To measure soil NO_3_^−^ and NH_4_^+^, 3 g of dry soil was extracted in 30-ml 2 M KCl for 1 hour before filtration (Whatman 42; 2.5-μm pore size). Soil NO_2_^−^ was extracted in water extracts (3 g of soil in 30 ml deionized water) to minimize its loss as gaseous N (*60*). Filtered extracts were analyzed using a colorimetric assay for NO_3_^−^ (SEAL method EPA-136-A), NH_4_^+^ (SEAL method EPA-129-A), and NO_2_^−^ (SEAL method EPA-137-A). Soil NO_3_^−^ and NH_4_^+^ were measured from all sites in June 2020, while soil NO_2_^−^ was measured from sites A, C, and D in July 2019.

### Field N_2_O emissions

An automated chamber system was used to sequentially measure N_2_O emissions from each of the collars under each of the eight shrubs. Each shrub was equipped with its own automated chamber (8100-104, LI-COR Biosciences, Lincoln, NE) connected to a multiplexer to automate the measurements (LI-8150, LI-COR Biosciences); chambers were measured sequentially so that fluxes were measured from each shrub every 30 min. While a given chamber was closed, gas was recirculated through a sample loop for 2 min. The sample loop connected the multiplexer to an infrared gas analyzer (LI-8100, LI-COR Biosciences) and an isotope N_2_O analyzer (Model 914-0027, Los Gatos Research Inc., Mountain View, CA). The instruments were kept in an air-conditioned box made from insulation boards (5 cm in thickness; 5 m by 2 m by 2 m; fig. S1). Occasional instrument errors prevented us from having a complete dataset. A water trap was also included in the sample loop to prevent condensation inside tubing lines fed to instruments during the transition from ambient conditions into the air-conditioned box. The infrared gas analyzer and N_2_O analyzer pulled air from the recirculating sample loop and vented the sampled air back into the sample loop; a vent in the chamber limited changes in chamber pressure (see the Supplementary Materials for full description of sample loop) (*61*). Diluting the sample loop with ambient air did not appreciably affect flux measurements because the amount of air entering the chamber over the relatively short 2-min measurement was small relative to the volume of the sample loop (~6 liters) and the change in N_2_O concentrations was linear (mean *R*^2^ = 0.80 when N_2_O flux is >1 ng of N-N_2_O m^−2^ s^−1^) throughout the measurements, especially when N_2_O emissions were high (mean *R*^2^ = 0.98 when N_2_O flux is >10 ng of N-N_2_O m^−2^ s^−1^) (*61*).

Field N_2_O emissions were calculated as the linear change in concentrations over the last 90 s of the 2-min incubation (*7*, *62*). Net emissions were reported as zero if the linear correlation between time and trace gas concentration was not statistically significant (*P* > 0.05). The isotopic N_2_O analyzer measured δ^15^N but because our measurements were diluted with ambient air, we did not attempt to calculate absolute δ^15^N values. Rather, for our field measurements, we calculated the average δ^15^N during the final 10 s of each incubation (hereafter referred to as *δ^15^N) and reported this as an index of the time it took the ^15^N tracer to be oxidized or reduced into N_2_O and detected by the analyzer.

## *narG* gene and transcript abundance

We extracted nucleic acids from ~2 g of soil collected underneath four shrubs from sites A and C in 2019 and site D in 2020. We did not sample site B because of limited resources; site B is relatively close to site A (fig. S1), so we omitted site B to maximize differences among sites. To ensure accurate capture of genes and transcripts, dry soils were collected right before starting field measurements, immediately frozen in liquid nitrogen in the field, and stored at −80°C until further processing. We first extracted RNA (QIAGEN RNeasy PowerSoil Total RNA kit) and then extracted DNA from the supernatant (PowerSoil DNA Elution Kit). To prepare nucleic acids for sequencing, DNA was removed from RNA extracts (RQ1 RNase-Free DNase; Promega) and reverse-transcribed into cDNA (ProtoScrip II Reverse Transcriptase; New England Biolabs). We used qPCR to estimate the abundance of *narG* and *napA* genes and transcripts, which encode for NO_3_^−^-reducing enzymes. We used the narG1960F/narG2650R primer set for *narG* (*63*) and the napA-V17m/napA4R primer set for *napA* (*35*). The 10-μl reactions consisted of 5 μl of a master-mix (Forget-Me-Not EvaGreen qPCR Master Mix; Biotium Inc., Fremont, CA), 0.8 μl of 2 mM MgCl_2_, 0.25 μl of bovine serum albumin (0.5 mg ml^−1^), 0.125 μl of 0.25 μM forward and reverse primer, 2.5 μl of H_2_O, and 1.2 μl of sample DNA. qPCR reactions were used to measure the quantity of *narG* and *napA* in RNA and DNA extracts (CFX384 Touch Real-Time PCR Detection System). All reactions were performed in triplicate. *narG* was amplified using the following protocol: 5 min at 95°C, followed by 40 cycles of 45 s at 95°C, 30 s at 50°C, and 60 s at 72°C. *napA* was amplified using the following protocol: 4 min at 95°C, followed by 40 cycles of 30 s at 95°C, 45 s at 65°C, and 60 s at 72°C.

We calculated the gene copy numbers per gram soil in each sample by running a standard curve in triplicate for each qPCR run. We synthesized known sequences of napA (​​National Center for Biotechnology Information reference sequence: NC_000913.3) and narG (NC_002945.4) as standards (gBlocks HiFi; Integrated DNA Technologies). We validated that the primers amplified the same size of PCR product in the standards and samples using gel electrophoresis. We prepared standard curves using serial dilutions for both narG (2 to 0.00002 ng/μl) and napA (10 to 0.00001 ng/μl). The *narG* standards had efficiencies of >65% (*R*^2^ = 0.99), and *napA* standards had efficiencies of >76% (*R*^2^ = 0.99).

### Chloroform inhibition experiment

To assess the relative contribution of biological and abiotic processes to N_2_O production, we slowed microbial activity with chloroform (CHCl_3_; an effective soil sterilant that slows the growth and recolonization of microbial communities resuscitating after wetting) (*64*) and compared N_2_O fluxes between CHCl_3_-fumigated and nonfumigated soils from site D in 2020—we chose this site because it produced the most N_2_O after wetting dry soils in the field, facilitating comparisons between fumigated and nonfumigated samples. Briefly, eight soil samples (~200 g; 0 to 10 cm in depth) were collected from underneath eight shrubs representative of our field measurements. From each of the eight samples, we took two duplicate 50 g of subsamples and placed them in mesocosms (0.12-liter canning jar); eight were left under ambient conditions in the laboratory, and the other eight were incubated in a vacuum-sealed chamber under a CHCl_3_ atmosphere for 10 days (*44*, *65*). Soils inside the incubation chamber were kept under a constant CHCl_3_ atmosphere by keeping a beaker full of 100 ml of liquid CHCl_3_ inside the chamber. To enhance the movement of CHCl_3_ into soil pores, we created a vacuum inside the chamber for 1 min and then allowed ambient air to flush into the chamber (*44*); this was repeated daily.

After 10 days under CHCl_3_, the mesocosms were removed from the chamber, and net N_2_O emissions were measured from fumigated and nonfumigated mesocosms over the course of an experimental wetting event. We also added ^15^N-NO_3_^−^ to the mesocosms to assess whether CHCl_3_ fumigation decreased the conversion of NO_3_^−^ to N_2_O. The ^15^N-NO_3_^−^ was dissolved in deionized water, and mesocosms were wetted with 10 ml of this solution (2 at % of ^15^N; 10 μg of N-NO_3_^−^ g^−1^ of dry soil). This volume increased gravimetric soil moisture to ~20%. We chose this water addition amount to approximate the upper limit of volumetric soil water content measured in response to wetting soils in the field; mean peak volumetric water content for each site ranged from 17 to 33%, where 30% volumetric water content is roughly equivalent to 20% gravimetric water content in these soils. Before wetting, mesocosms were placed in a 40°C water bath to simulate summer temperatures at site D. To measure net N_2_O emissions during the incubation, the headspace from each mesocosm was dried using a Nafion dryer (PD-200 T-12MPS, Perma Pure LLC, Lakewood Township, NJ, USA) and recirculated through a sample loop connected to a multiplexer (LI-8150, LI-COR Biosciences) and an isotope N_2_O analyzer (Model 914-0027, Los Gatos Research Inc., Mountain View, CA). Gas was recirculated through the closed sample loop at a rate of 1.5 liter min^−1^. Net N_2_O emissions were calculated as the linear change in N_2_O concentration over the 2-min incubation period. After recirculating and measuring the air from one mesocosm for 2 min, the multiplexer flushed the sample loop with room air for 2 min and then sampled the next mesocosm in the sequence; four mesocosms were connected to the multiplexer at once, meaning that each mesocosm was measured every 16 min ([2-min measurement + 2-min flush] × 4 replicates). N_2_O measurements for each mesocosm began 5 min before wetting and were measured every 16 min for at least 8 hours after wetting. While the recirculation of sample air likely dried out soils throughout the incubation, this is consistent with the drying of soils in the field after wetting (fig. S8). The δ^15^N^bulk^ emitted from soil was measured using Keeling plots (*12*, *66*); δ^15^N^bulk^ was calculated as the intercept when plotting the inverse of soil N_2_O concentrations on the *x* axis versus measured δ^15^N on the *y* axis. We corrected δ^15^N^bulk^ values for known N_2_O and CO_2_ mass dependencies using instrument-specific calibration curves developed using established methods (*27*). The calibration curves were created by analyzing δ^15^N^bulk^ of a certified standard referenced against U.S. Geological Survey (USGS) 51 and 52 isotope reference materials (Reston Stable Isotope Laboratory, Reston, VA, USA), while varying N_2_O concentration (between 0.3 and 5 ppm) across three different CO_2_ concentrations (330, 660, and 990 ppm).

### Natural abundance N_2_O isotope laboratory experiment

We conducted a second laboratory incubation experiment to investigate the processes producing N_2_O in soils from site D using the natural abundance isotopic composition of N_2_O (SP, δ^15^N^bulk^, and δ^18^O) over the course of an experimental wetting event. We chose site D because it consistently produced the most N_2_O after wetting dry soils in the field, allowing us to maximize our ability to characterize the N_2_O. The isotopic composition of N_2_O was measured after adding water to air-dried soils (50 g; *n* = 6) to raise the gravimetric water content to 20% (fig. S8). Soils were incubated in closed mesocosms (0.12-liter glass canning jar) at 40°C; each mesocosm was purged with zero air and connected to a 1-liter foil gas bag (Cali-5-Bond, Calibrated Instruments LLC; McHenry, MD) filled with zero air for the duration of the incubation (*26*). Following the 6-hour incubation, gas from the mesocosm headspace and gas bag was thoroughly mixed by pumping the mesocosm headspace for one minute with a 60-ml syringe. After mixing, the gas bag was detached from the mesocosm and attached to the N_2_O isotope analyzer (described above) for analysis.

The N_2_O isotope analyzer was set to withdraw sample air from each 1-liter gas bag at 80 ml min^−1^ for ~12 min, recording N_2_O concentrations and isotope values every second. To avoid interferences caused by CO_2_, volatile organic compounds, and water vapor on N_2_O isotope measurements, the gas passed through a CO_2_ trap (Carbosorb, Elemental Microanalysis, Okehampton, UK), a volatile organic compound trap (silica gel and activated charcoal, Sigma-Aldrich, St. Louis, MO, USA), and a Nafion water trap (PD-200 T-12MPS, Perma Pure LLC, Lakewood Township, NJ, USA) before entering the N_2_O analyzer (*26*). To calculate SP, δ^15^N^bulk^, and δ^18^O, we averaged the last ~3 min of our gas bag measurements, where each gas bag was measured every second for a total of 12 min. We corrected our data using a standard curve made with USGS 51 (δ^15^N^bulk^ = 1.32‰, δ^15^N^α^ = 0.48, δ^15^N^β^ = 2.15, SP = −1.67‰, δ^18^O = 41.23‰) and USGS 52 (δ^15^N^bulk^ = 0.44‰, δ^15^N^α^ = 13.52, δ^15^N^β^ = −12.64, SP = 26.15‰, δ^18^O = 40.64‰) N_2_O isotope reference materials (Reston Stable Isotope Laboratory, Reston, Virginia, USA). Individual standard curves were made for three isotopocules of N_2_O: ^15^N^14^N^16^O, ^14^N^15^N^16^O, and ^14^N^14^N^18^O (*26*, *67*). The standard curves were highly linear (*R*^2^ > 0.99) between 0.6 and 8 ppm of N_2_O. The corrected concentration of each isotopocule was converted into delta notation for interpretation using the following equations (*26*)

$$\delta^{15}N^{\alpha }=\left[\frac{{\left(N^{15}\mathrm{NO}/N_{2}O\right)}_{\mathrm{sample}}}{{\left(N^{15}\mathrm{NO}/N_{2}O\right)}_{\mathrm{std}}}-1\right]*1000$$

$$\delta^{15}N^{\beta }=\left[\frac{{\left({}^{15}\mathrm{NNO}/N_{2}O\right)}_{\mathrm{sample}}}{{\left({}^{15}\mathrm{NNO}/N_{2}O\right)}_{\mathrm{std}}}-1\right]*1000$$

$$\delta^{18}O=\left[\frac{{\left(NN^{18}O/N_{2}O\right)}_{\mathrm{sample}}}{{\left(NN^{18}O/N_{2}O\right)}_{\mathrm{std}}}-1\right]*1000$$

We calculated SP as the difference between δ^15^N^α^ and δ^15^N^β^

$$\mathrm{SP}=\delta^{15}N^{\alpha }-\delta^{15}N^{\beta }$$

As a measure of uncertainty, averaging 1-s values for 3 min (*n* = 180) at the N_2_O concentration range of our samples [630 to 8072 parts per billion (ppb)] produced coefficients of variation <2.9% for all measured isotopes (table S2).

### Statistical analyses

All statistical analyses were conducted using R 4.2.2 (*68*). Linear regression was used to determine whether adding either NO_3_^−^ or NH_4_^+^ increased N_2_O emissions from each site. For each linear model, peak soil N_2_O emissions from each shrub were included as the response variable, and the amount of N was added as the predictor variable; separate models were run for NO_3_^−^ and NH_4_^+^ at each site. Peak N_2_O emissions were calculated as the highest emission from a given collar over the 24 hours after wetting. We used peak N_2_O emissions rather than cumulative N_2_O emissions because there were missing observations from sites C and D (due to instrument malfunction) that limited our ability to integrate the area under the curve. Using a prior dataset collected using similar methods (*7*), we found a strong positive linear relationship between cumulative and peak N_2_O emissions (*R*^2^ = 0.95, *P* < 0.001), justifying this approach. If peak N_2_O emissions did not follow a normal distribution (as assessed using a Shapiro-Wilk test), then log transformations were applied. We expected peak N_2_O emissions and N addition amount to be related linearly because nitrate is the primary limiting substrate for denitrification (*4*). However, we tested for nonlinear relationships between N addition amount and peak N_2_O emissions using the nlcor package in R (*69*) but did not detect any significant relationships (*P* > 0.10). We used analysis of variance (ANOVA) to assess whether *narG* and *napA* gene and transcript copy number differed between sites A, C, and D. If the ANOVA was statistically significant (*P* < 0.05), then we used Tukey corrected multiple comparisons to assess differences between sites. Last, we used a paired *t* test to determine whether adding CHCl_3_ decreased cumulative N_2_O emissions during the CHCl_3_ microbial sterilization laboratory experiment.

## Supplementary Materials

### This PDF file includes:

Supplemental Methods Tables S1 to S3 Figs. S1 to S8 References
