# Supplementary material for: Bacterial denitrification drives elevated N2O emissions in arid southern California drylands
Source: Sci Adv. 2023 Dec 6;9(49):eadj1989. doi: 10.1126/sciadv.adj1989 (PMC10699777; doi:10.1126/sciadv.adj1989)
Supplement: Supplementary file 1 — Supplemental Methods Tables S1 to S3 Figs. S1 to S8 References [file sciadv.adj1989_sm.pdf]

Science Advances
AAAS

Supplementary Materials for

Bacterial denitrification drives elevated N_2O emissions in arid southern California drylands

Alexander H. Krichels *et al.*

Corresponding author: Alexander H. Krichels, alexander.krichels@ucr.edu, alexander.krichels@usda.gov

Sci. Adv. **9**, eadj1989 (2023)
DOI: 10.1126/sciadv.adj1989

This PDF file includes:

Supplemental Methods
Tables S1 to S3
Figs. S1 to S8
References

Supplemental Methods

Soil nitric oxide (NO) emissions.

A nitric oxide (NO) analyzer (Model 410 and Model 401, 2B Technologies, Boulder CO) was connected to the field sample loop (described in section 2.3 of the manuscript) for the 2019 field campaign. The NO analyzer sampled air from the recirculating sample loop at a rate of 1 L min^{-1} and vented the air to the atmosphere. A vent in the chamber allowed for ambient air to enter the sample loop to prevent changes in chamber pressure (*61*). Field NO emissions were calculated as the linear change in concentrations over the last 90 seconds of the two-minute incubation (*7*, *62*). Net emissions were reported as zero if the linear correlation between time and trace gas concentration was not statistically significant ($p > 0.05$). The change in NO concentrations was linear (mean $R^2 = 0.86$ for all measurements) especially when NO emissions were high (mean $R^2 = 0.98$ when emissions > 100 ng N-NO m^{-2} s^{-1}).

We also measured the conversion of ^{15}N-NO_3^- and ^{15}N-NH_4^+ to NO from all four sites in June 2020 using passive samplers (Ogawa pads; Ogawa USA, Pompano Beach, FL). Four separate pairs of collars were installed under four different shrubs. One collar was wet with ^{15}N-NO_3^- solution on the first day of the experiment, and the other was wet with ^{15}N-NH_4^+ solution on the second day of the experiment. Passive NO_x and NO_2 sampling pads were installed within closed chambers for two discrete time periods post wetting: 0–15 minutes and 15 minutes–24 hours. The chambers were sealed with a rubber lid to prevent gas from escaping during the incubation. The passive sampling pads were stored in plastic bags and transported to the lab to measure NO_x concentrations and isotopic composition. The NO_x from the pads was extracted as NO_2^- in 8 mL deionized water and the solution was analyzed for NO_2^- concentrations (SEAL method EPA-129-A); we did not detect NO_2^- on the NO_2 pads, suggesting the NO_x pads

exclusively collected NO. $\delta^{15}N$-NO composition was then measured by converting the extracted NO_2^- to N_2O using *Pseudomonas aureofaciens* (*73*) and analyzing the N_2O for $\delta^{15}N$ using a Thermo Delta V isotope ratio mass spectrometer (Thermo Fisher Scientific, Woltham, MA) at the Facility for Isotope Ratio Mass Spectrometry (FIRMS; https:// ccb.ucr.edu/facilities/firms) at the University of California, Riverside.

Soil Chemical Properties

We measured extractable NO_3^- and NH_4^+ concentrations and pH in soils collected from underneath (0–10 cm depth) each shrub prior to our wetting experiments. Briefly, 5 g soil samples were shaken in 2M KCl (30 mL) for one hour, filtered (Whatman 42 filter paper; 2.5 µm pore size), and frozen until analysis. Colorimetric assays were used to measure NH_4^+ (SEAL method EPA-126-A) and NO_3^- (SEAL method EPA-129-A) in the KCl extracts. Soil total C and N were measured in an elemental analyzer (Flash EA1112; Thermo Scientific, Woltham, MA) at the Environmental Sciences Research Laboratory at the University of California, Riverside (https://envisci.ucr.edu/research/environmental-sciences-research-laboratory-esrl). Soil pH was measured in a 1:1 soil to water ratio with a pH meter (Orion VersaStar Pro; Thermo Scientific, Woltham, MA).

Table S1. Soil taxonomic classification at our study sites (Soil Survey Staff, 2022).

Site	Taxonomic Class	Series	Texture
A) Morongo	Thermic Fluventic Haploxerolls	Gorgonio Series	Gravelly loamy fine sand
B) Oasis	Thermic Typic Xeropsamments	Tujunga Series	Gravelly loamy sand
C) Wide Canyon	Mixed, Hyperthermic Typic Torripsamments	Carsitas Series	Gravelly sand
D) Pinto Basin	Thermic Typic Haplargids	Cajon-Friedliver Complex	Gravelly Sand

Table S2. Isotopic composition of lab incubated soils from site D. Values represent the mean ± standard deviation over the last 3 minutes (n = 180 datapoints) used to analyze each replicate on the N_2O laser. SP was calculated as the difference between $\delta^{15}N^{\alpha}$ and $\delta^{15}N^{\beta}$, while $\delta^{15}N^{bulk}$ was calculated as the mean of $\delta^{15}N^{\alpha}$ and $\delta^{15}N^{\beta}$.

Rep	N_2O (ppb)	$\delta^{15}N^{\alpha}$ (‰)	$\delta^{15}N^{\beta}$ (‰)	$\delta^{18}O$ (‰)
1	2288 ± 3.85	11.8 ± 0.048	-1.50 ± 0.008	49.7 ± 0.40
2	1130 ± 1.54	36.9 ± 0.28	22.03 ± 0.24	51.4 ± 0.91
3	1788 ± 4.76	16.2 ± 0.084	4.45 ± 0.032	49.0 ± 0.54
4	633 ± 0.077	32.3 ± 0.40	14.9 ± 0.28	48.5 ± 1.39
5	1430 ± 2.96	38.6 ± 0.23	24.9 ± 0.20	52.0 ± 0.68
6	8072 ± 20.2	14.5 ± 0.043	8.71 ± 0.026	34.7 ± 0.16

Table S3. Linear relationships between the amount of added NO_3^- or NH_4^+ (kg N ha^{-1}) and peak N_2O emissions (µg N m^{-2}) over the course of 24 h from each field site. Slope, intercept, and marginal R^2 are only reported when $p < 0.05$.

Time	N treatment	Site	Slope	Intercept	p-value	n	R^2
July 2019	NO_3^-	A	--	--	0.51	8	--
		C	--	--	0.71	8	--
		D	5.32	-34.2	**0.0082**	8	0.67
	NH_4^+	A	--	--	0.58	8	--
		C	--	--	0.24	8	--
June 2020	NO_3^-	A	--	--	0.26	8	--
		B	--	--	0.20	8	--
		C	--	--	0.65	8	--
		D	--	--	0.89	8	--
	NH_4^+	A	--	--	0.65	8	--
		B	--	--	0.39	8	--
		C	--	--	0.92	8	--
		D	--	--	0.19	8	--
August 2020	NO_3^-	A	--	--	0.95	8	--
		B	--	--	0.77	8	--
		C	--	--	0.25	8	--
		D	15.0	-30.1	**0.036**	8	0.47
	NH_4^+	A	--	--	0.50	8	--
		B	--	--	0.13	8	--
		C	--	--	0.066	8	--
		D	--	--	0.12	8	--

Supplemental Figures

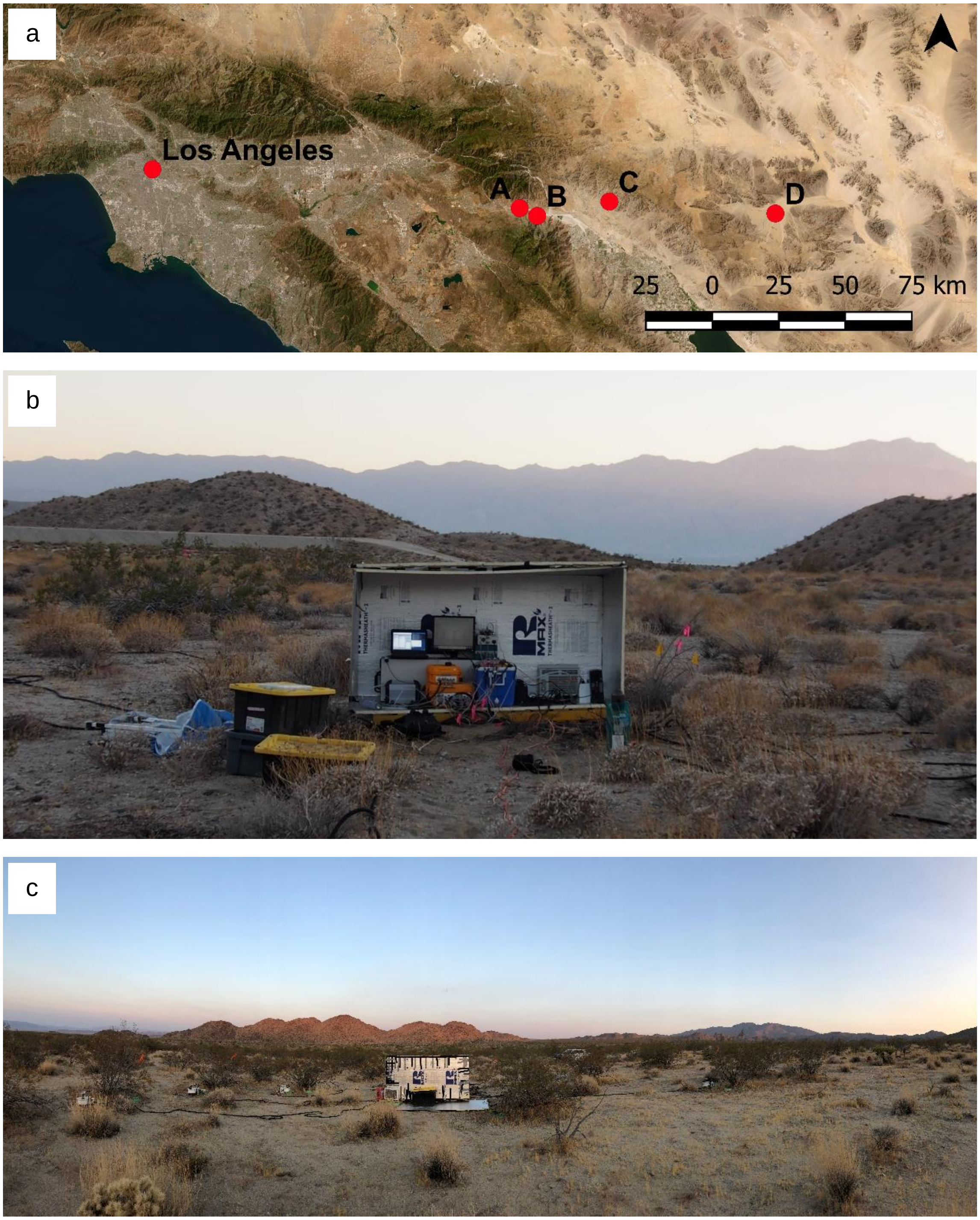

Figure S1. Map of the four study sites (a), photo of instrument setup at site C (b) and photo of instrument setup at site D (c).

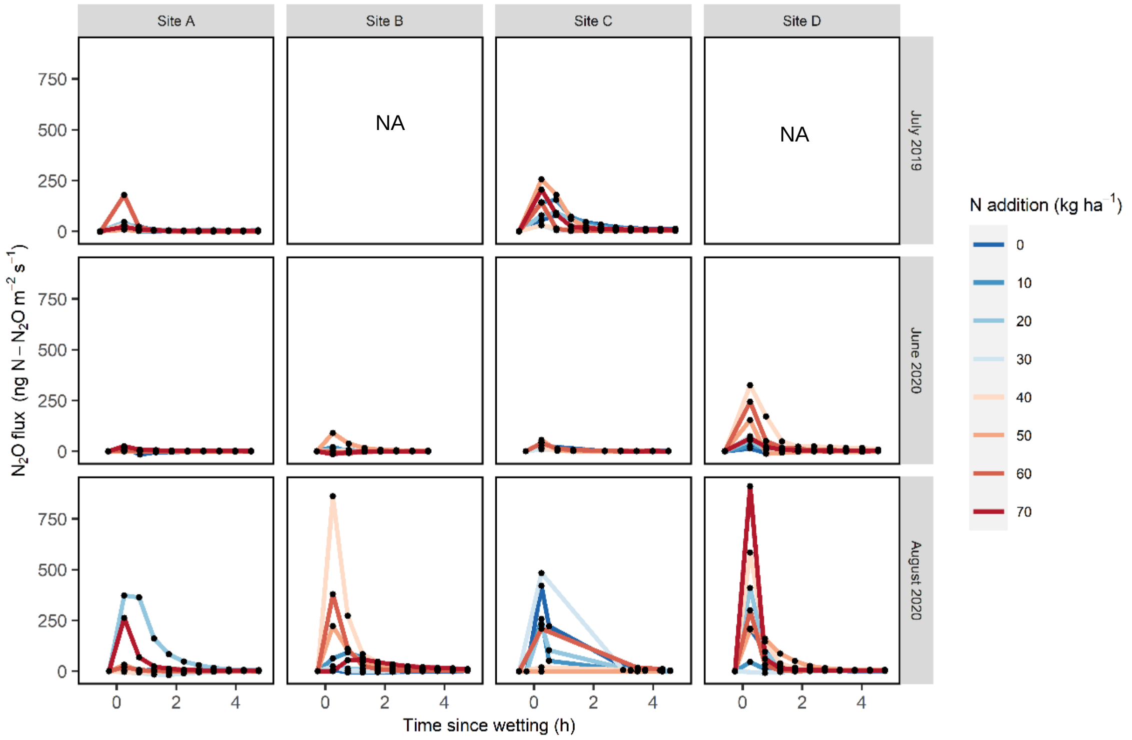

Figure S2. Field N_2O emissions (ng N-N_2O m^{-2} s^{-1}) over five hours after wetting summer-dry soils with ammonium solutions. Each black dot represents flux measurements over a 2-minute period for each of the eight automated chambers under N treatment (line colors correspond to levels of N enrichment; kg ha^{-1}). NA = data not available; see methods.

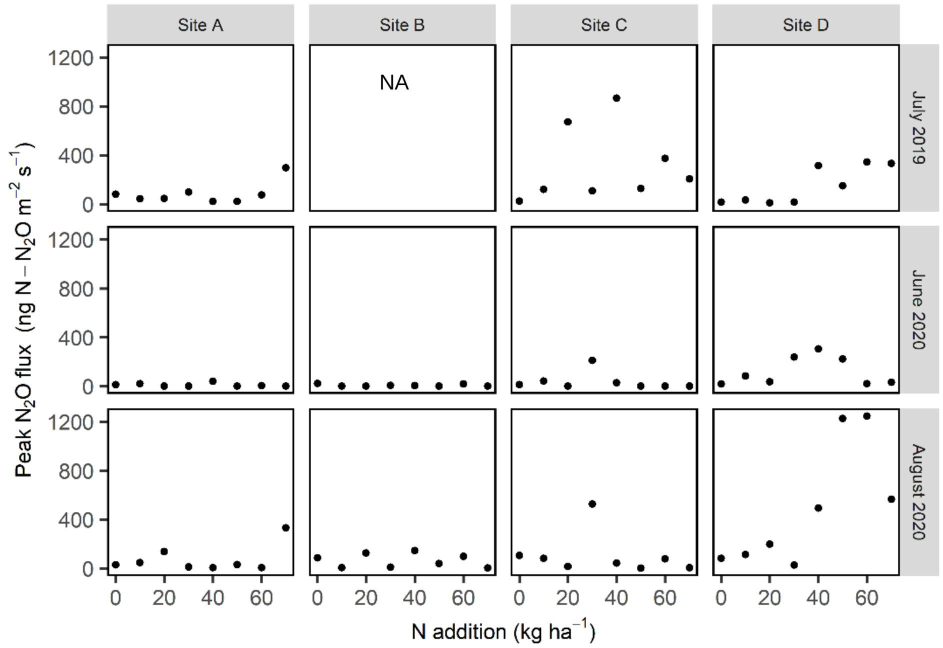

Figure S3. Peak N_2O emissions (ng N-N_2O m^{-2} s^{-1}) after wetting summer-dry soils with nitrate solutions. Each black dot represents the highest N_2O flux measurement over the 24 hours after adding nitrate solutions to soil collars (x-axis corresponds to NO_3^- addition amount; kg ha^{-1}). NA = data not available; see methods.

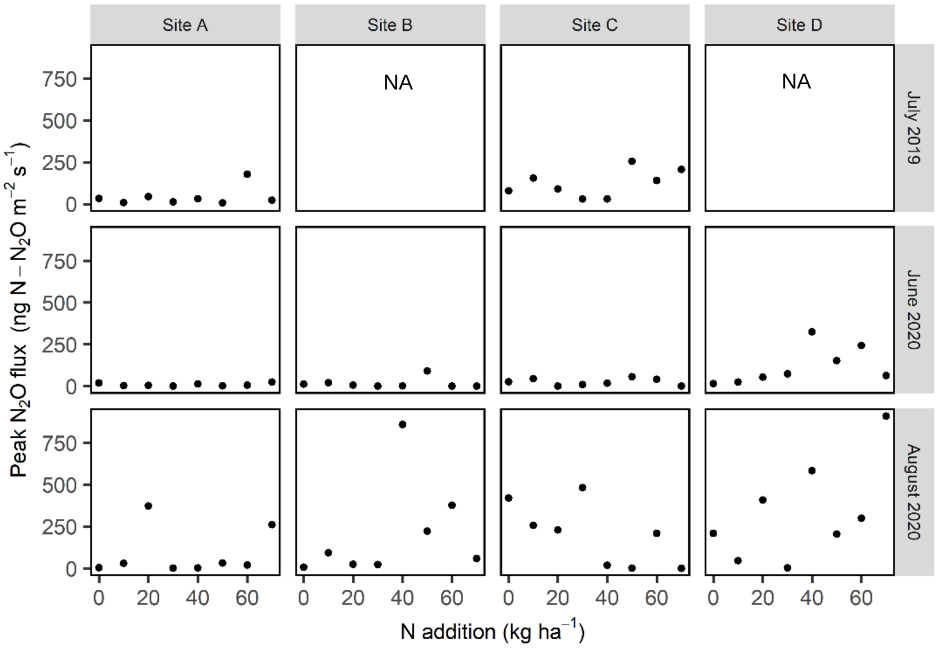

Figure S4. Peak N_2O emissions (ng N-N_2O m^{-2} s^{-1}) after wetting summer-dry soils with ammonium solutions. Each black dot represents the highest N_2O flux measurement over the 24 hours after adding ammonium solutions to soil collars (x-axis corresponds to NH_4^+ addition amount; kg ha^{-1}). NA = data not available; see methods.

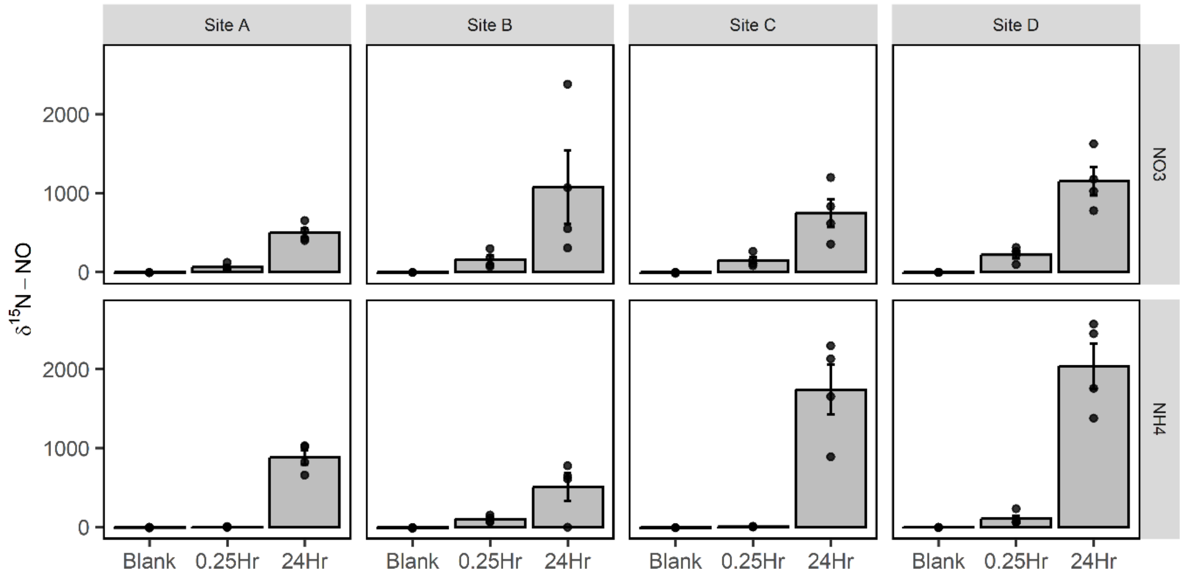

Figure S5. Isotope values for δ^{15}N-NO emitted from each site 15 minutes and 24 hours after wetting dry soils with nitrate (top row) or ammonium (bottom row) solutions in 2020. Bars represent the mean δ^{15}N-NO (n = 4) from each treatment within each site, error bars represent standard errors, and dots represent individual measurements.

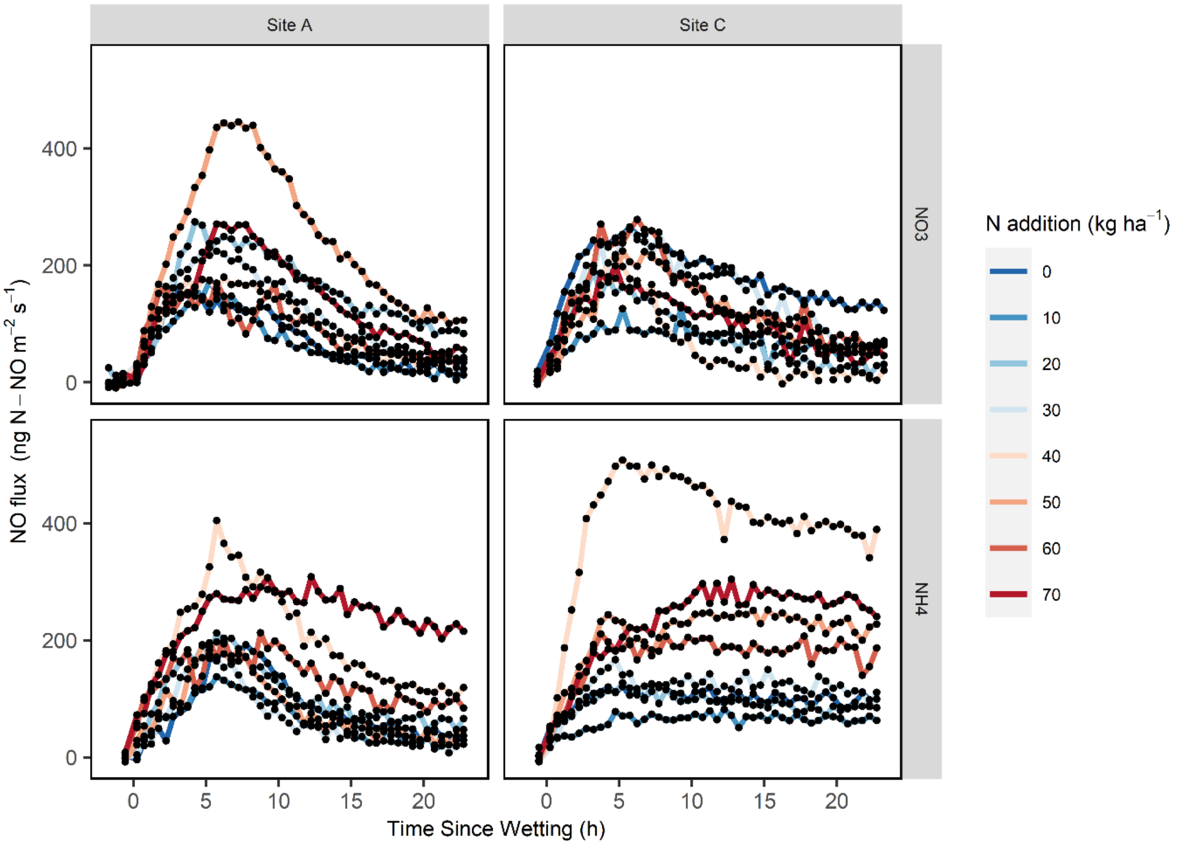

Figure S6. Field NO emissions (ng N-NO m^{-2} s^{-1}) over 24 hours from site A (a, c) and site C (b, d) after wetting summer-dry soils with nitrate (a, b) or ammonium (c, d) solutions. Each black dot represents flux measurements over a 2-minute period for each of the eight automated chambers under N treatment (line colors correspond to levels of N enrichment; kg ha^{-1}).

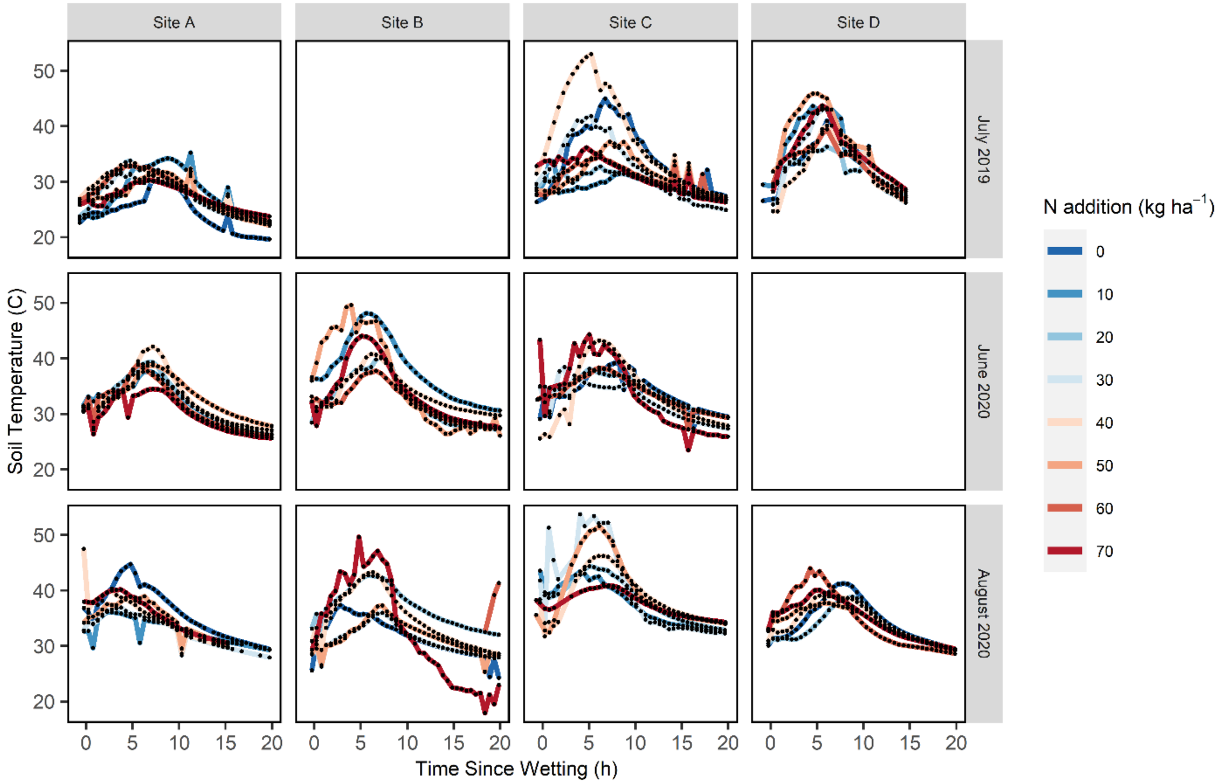

Figure S7. Soil temperature (°C) from sites A, B, C, and D after wetting summer-dry soils with nitrate solutions. Each black dot represents the average soil temperature over a 2-minute period for each of the eight automated chambers under N treatment (line colors correspond to levels of N enrichment; kg ha^{-1}).

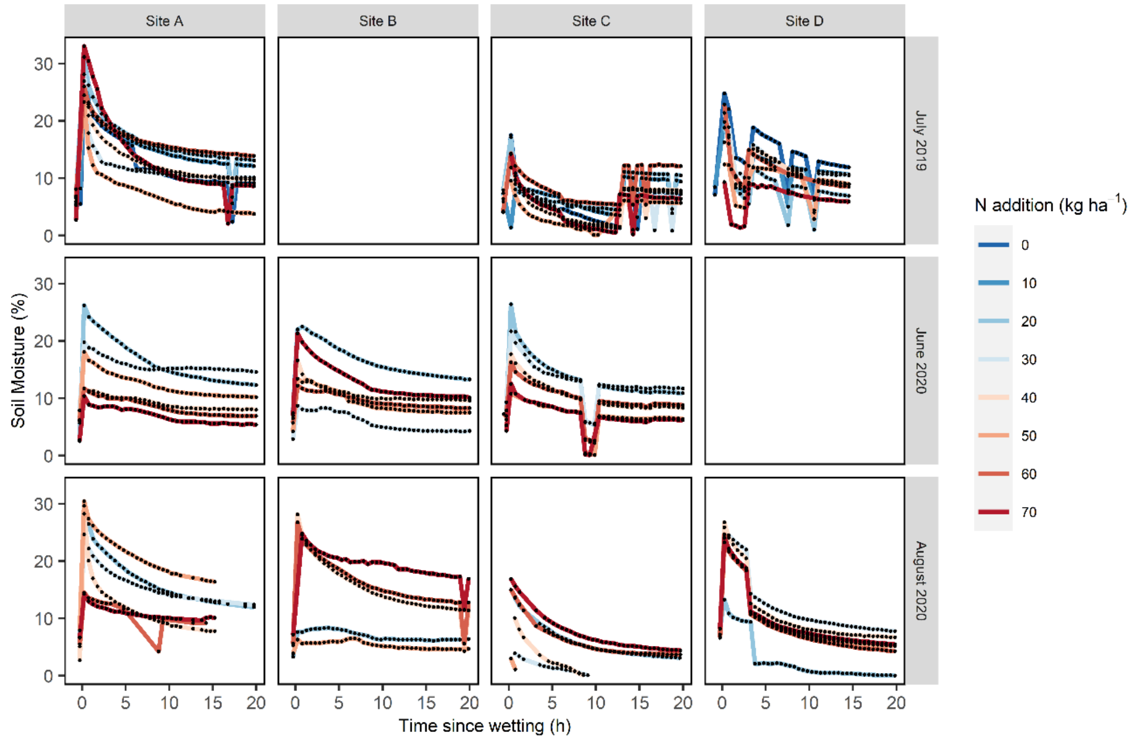

Figure S8. Volumetric soil water content (%) from sites A, B, C, and D after wetting summer-dry soils with nitrate solutions. Each black dot represents the average soil moisture over a 2-minute period for each of the eight automated chambers under N treatment (line colors correspond to levels of N enrichment; kg ha^{-1}).

REFERENCES

1. A. R. Ravishankara, J. S. Daniel, R. W. Portmann, Nitrous oxide (N_2O): The dominant ozone-depleting substance emitted in the 21st century. *Science* **326**, 123–125 (2009).

2. H. Tian, R. Xu, J. G. Canadell, R. L. Thompson, W. Winiwarter, P. Suntharalingam, E. A. Davidson, P. Ciais, R. B. Jackson, G. Janssens-Maenhout, M. J. Prather, P. Regnier, N. Pan, S. Pan, G. P. Peters, H. Shi, F. N. Tubiello, S. Zaehle, F. Zhou, A. Arneth, G. Battaglia, S. Berthet, L. Bopp, A. F. Bouwman, E. T. Buitenhuis, J. Chang, M. P. Chipperfield, S. R. S. Dangal, E. Dlugokencky, J. W. Elkins, B. D. Eyre, B. Fu, B. Hall, A. Ito, F. Joos, P. B. Krummel, A. Landolfi, G. G. Laruelle, R. Lauerwald, W. Li, S. Lienert, T. Maavara, M. MacLeod, D. B. Millet, S. Olin, P. K. Patra, R. G. Prinn, P. A. Raymond, D. J. Ruiz, G. R. van der Werf, N. Vuichard, J. Wang, R. F. Weiss, K. C. Wells, C. Wilson, J. Yang, Y. Yao, A comprehensive quantification of global nitrous oxide sources and sinks. *Nature* **586**, 248–256 (2020).

3. P. Forster, T. Storelvmo, K. Armour, W. Collins, J. L. Dufresne, D. Frame, D. J. Lunt, T. Mauritsen, M. D. Palmer, M. Watanabe, M. Wild, H. Zhang, The Earth’s Energy Budget, Climate Feedbacks, and Climate Sensitivity, in *Climate Change 2021: The Physical Science Basis. Contribution of Working Group I to the Sixth Assessment Report of the Intergovernmental Panel on Climate Change*, V. Masson-Delmotte, P. Zhai, A. Pirani, S. L. Connors, C. Péan, S. Berger, N. Caud, Y. Chen, L. Goldfarb, M. I. Gomis, M. Huang, K. Leitzell, E. Lonnoy, J. B. R. Matthews, T. K. Maycock, T. Waterfield, O. Yelekçi, R. Yu, B. Zhou, Eds. (Cambridge University Press, Cambridge, UK and New York), pp. 923–1054 (2001).

4. R. Knowles, Denitrification. *Microbiol. Rev.* **46**, 43–70 (1982).

5. A. J. Burgin, W. H. Yang, S. K. Hamilton, W. L. Silver, Beyond carbon and nitrogen: How the microbial energy economy couples elemental cycles in diverse ecosystems. *Front. Ecol. Environ.* **9**, 44–52 (2011).

6. J. P. Megonigal, M. E. Hines, P. T. Visscher, Anaerobic metabolism: Linkages to trace gases and aerobic processes, in *Treatise on Biogeochemistry*, W. H. Schlesinger, Ed. (Elsevier-Pergamon, 2003), pp. 317–424.

7. A. H. Krichels, P. M. Homyak, E. L. Aronson, J. O. Sickman, J. Botthoff, H. Shulman, S. Piper, H. M. Andrews, G. D. Jenerette, Rapid nitrate reduction produces pulsed NO and N_2O emissions following wetting of dryland soils. *Biogeochemistry* **158**, 233–250 (2022).

8. J. R. Eberwein, P. M. Homyak, C. J. Carey, E. L. Aronson, G. D. Jenerette, Large nitrogen oxide emission pulses from desert soils and associated microbiomes. *Biogeochemistry* **149**, 239–250 (2020).

9. T. Zhao, A. Dai, CMIP6 model-projected hydroclimatic and drought changes and their causes in the twenty-first century. *J. Climate* **35**, 897–921 (2022).

10. M. K. Firestone, E. A. Davidson, Microbiological basis of NO and N_2O production and consumption in soil, in *Exchange of Trace Gases between Terrestrial Ecosystems and the Atmosphere* (John Wiley and Sons, New York, 1989), pp. 7–21.

11. W. T. Peterjohn, Denitrification: Enzyme content and activity in desert soils. *Soil Biol. Biochem.* **23**, 845–855 (1991).

12. H. A. Barrat, I. M. Clark, J. Evans, D. R. Chadwick, L. Cardenas, The impact of drought length and intensity on N cycling gene abundance, transcription and the size of an N_2O hot moment from a temperate grassland soil. *Soil Biol. Biochem.* **168**, 108606 (2022).

13. F. Hafeez, J. C. Clément, L. Bernard, F. Poly, T. Pommier, Early spring snowmelt and summer droughts strongly impair the resilience of bacterial community and N cycling functions in a subalpine grassland ecosystem. *Oikos* **2023**, e09836 (2023).

14. T. Pérez, S. E. Vergara, W. L. Silver, Assessing the climate change mitigation potential from food waste composting. *Sci. Rep.* **13**, 7608 (2023).

15. J. Heil, H. Vereecken, N. Brüggemann, A review of chemical reactions of nitrification intermediates and their role in nitrogen cycling and nitrogen trace gas formation in soil. *Eur. J. Soil Sci.* **67**, 23–39 (2016).

16. X. Zhu-Barker, A. R. Cavazos, N. E. Ostrom, W. R. Horwath, J. B. Glass, The importance of abiotic reactions for nitrous oxide production. *Biogeochemistry* **126**, 251–267 (2015).

17. P. M. Homyak, J. C. Blankinship, K. Marchus, D. M. Lucero, J. O. Sickman, J. P. Schimel, Aridity and plant uptake interact to make dryland soils hotspots for nitric oxide (NO) emissions. *Proc. Natl. Acad. Sci. U.S.A.* **113**, E2608–E2616 (2016).

18. M. Ermel, T. Behrendt, R. Oswald, B. Derstroff, D. Wu, S. Hohlmann, C. Stönner, A. Pommerening-Röser, M. Könneke, J. Williams, F. X. Meixner, M. O. Andreae, I. Trebs, M. Sörgel, Hydroxylamine released by nitrifying microorganisms is a precursor for HONO emission from drying soils. *Sci. Rep.* **8**, 1–8 (2018).

19. E. A. Davidson, Sources of nitric oxide and nitrous oxide following wetting of dry soil. *Soil Sci. Soc. Am. J.* **56**, 95–102 (1992).

20. E. Harris, E. Diaz-Pines, E. Stoll, M. Schloter, S. Schulz, C. Duffner, K. Li, K. L. Moore, J. Ingrisch, D. Reinthaler, S. Zechmeister-Boltenstern, S. Glatzel, N. Brüggemann, M. Bahn, Denitrifying pathways dominate nitrous oxide emissions from managed grassland during drought and rewetting. *Science Sci. Adv.* **7**, eabb7118 (2021).

21. E. A. Davidson, J. Chorover, D. B. Dail, A mechanism of abiotic immobilization of nitrate in forest ecosystems: The ferrous wheel hypothesis. *Glob. Chang. Biol.* **9**, 228–236 (2003).

22. F. Matus, S. Stock, W. Eschenbach, J. Dyckmans, C. Merino, F. Nájera, M. Köster, Y. Kuzyakov, M. A. Dippold, Ferrous wheel hypothesis: Abiotic nitrate incorporation into dissolved organic matter. *Geochim. Cosmochin. Acta.* **245**, 514–524 (2019).

23. B. P. Colman, N. Fierer, J. P. Schimel, Abiotic nitrate incorporation in soil: Is it real? *Biogeochemistry* **84**, 161–169 (2007).

24. B. P. Colman, N. Fierer, J. P. Schimel, Abiotic nitrate incorporation, anaerobic microsites, and the ferrous wheel. *Biogeochemistry* **91**, 223–227 (2008).

25. L. Yu, E. Harris, D. Lewicka-Szczebak, M. Barthel, M. R. A. Blomberg, S. J. Harris, M. S. Johnson, M. F. Lehmann, J. Liisberg, C. Müller, N. E. Ostrom, J. Six, S. Toyoda, N. Yoshida, J. Mohn, What can we learn from N_2O isotope data? – Analytics, processes and modelling. *Rapid Commun. Mass Spectrom.* **34**, e8858. (2020).

26. E. R. Stuchiner, Z. D. Weller, J. C. von Fischer, An approach for calibrating laser-based N_2O isotopic analyzers for soil biogeochemistry research. *Rapid Commun. Mass Spectrom.* **35**, e8978 (2021).

27. S. J. Harris, J. Liisberg, L. Xia, J. Wei, K. Zeyer, L. Yu, M. Barthel, B. Wolf, B. F. J. Kelly, D. I. Cendón, T. Blunier, J. Six, J. Mohn, N_2O isotopocule measurements using laser spectroscopy: Analyzer characterization and intercomparison. *Atmos. Meas. Tech.* **13**, 2797–2831 (2020).

28. S. D. Wankel, W. Ziebis, C. Buchwald, C. Charoenpong, D. de Beer, J. Dentinger, Z. Xu, K. Zengler, Evidence for fungal and chemodenitrification based N_2O flux from nitrogen impacted coastal sediments. *Nat. Commun.* **8**, 15595 (2017).

29. J. Wei, E. Ibraim, N. Brüggemann, H. Vereecken, J. Mohn, First real-time isotopic characterisation of N_2O from chemodenitrification. *Geochim. Cosmochim. Acta* **267**, 17–32 (2019).

30. R. M. M. Abed, P. Lam, D. De Beer, P. Stief, High rates of denitrification and nitrous oxide emission in arid biological soil crusts from the Sultanate of Oman. *ISME J.* **7**, 1862–1875 (2013).

31. J. Shapleigh, Denitrifying prokaryotes, in *The Prokaryotes*, E. Rosenbers, Ed. (Springer-Verlag, ed. 4, 2013), pp. 405–425.

32. S. Maier, A. M. Kratz, J. Weber, M. Prass, F. Liu, A. T. Clark, R. M. M. Abed, H. Su, Y. Cheng, T. Eickhorst, S. Fiedler, U. Pöschl, B. Weber, Water-driven microbial nitrogen transformations in biological soil crusts causing atmospheric nitrous acid and nitric oxide emissions. *ISME J.* **16**, 1012–1024 (2022).

33. C. Moreno-Vivián, P. Cabello, M. Martínez-Luque, R. Blasco, F. Castillo, Prokaryotic nitrate reduction: Molecular properties and functional distinction among bacterial nitrate reductases. *J. Bacteriol.* **181**, 6573–6584 (1999).

34. X. Chen, C. Liu, B. Zhu, W. Wei, R. Sheng, The contribution of nitrate dissimilation to nitrate consumption in *narG*- and *napA*-containing nitrate reducers with various oxygen and nitrate supplies. *Microbiol. Spectr.* **10**, e0069522 (2022).

35. D. Bru, A. Sarr, L. Philippot, Relative abundances of proteobacterial membrane-bound and periplasmic nitrate reductases in selected environments. *Appl. Environ. Microbiol.* **73**, 5971–5974 (2007).

36. E. M. Lacroix, J. Mendillo, A. Gomes, A. Dekas, S. Fendorf, Contributions of anoxic microsites to soil carbon protection across soil textures. *Geoderma* **425**, 116050 (2022).

37. F. Garcia-Pichel, J. Belnap, Microenvironments and microscale productivity of cyanobacterial desert CRUSTS[1]. *J. Phycol.* **32**, 774–782 (1996).

38. A. J. Sexstone, N. P. Revsbech, T. B. Parkin, J. M. Tiedje, Direct measurement of oxygen profiles and denitrification rates in soil aggregates. *Soil Sci. Soc. Am. J.* **49**, 645–651 (1985).

39. E. M. Lacroix, R. J. Rossi, D. Bossio, S. Fendorf, Effects of moisture and physical disturbance on pore-scale oxygen content and anaerobic metabolisms in upland soils. *Sci. Total Environ.* **780** (2021), 146572.

40. H. M. Andrews, A. H. Krichels, P. M. Homyak, S. Piper, E. L. Aronson, J. Botthoff, A. C. Greene, G. D. Jenerette, Wetting-induced soil CO_2 emission pulses are driven by interactions among soil temerature, carbon, and nitrogen limitation in the Colorado Desert. *Glob. Chang. Biol.* **29**, 3205–3220 (2023).

41. Y. Du, S. Guo, R. Wang, X. Song, X. Ju, Soil pore structure mediates the effects of soil oxygen on the dynamics of greenhouse gases during wetting-drying phases. *Sci. Total Environ.* **895**, 165192 (2023).

42. E. Bueno, D. Mania, Å. Frostegard, E. J. Bedmar, L. R. Bakken, M. J. Delgado, Anoxic growth of *Ensifer meliloti* 1021 by N_2O-reduction, a potential mitigation strategy. *Front. Microbiol.* **6**, 1–11 (2015).

43. L. Bergaust, Y. Mao, L. R. Bakken, Å. Frostegård, Denitrification response patterns during the transition to anoxic respiration and posttranscriptional effects of suboptimal pH on nitrogen oxide reductase in *Paracoccus denitrificans*. *Appl. Environ. Microbiol.* **76**, 6387–6396 (2010).

44. J. C. Blankinship, C. A. Becerra, S. M. Schaeffer, J. P. Schimel, Separating cellular metabolism from exoenzyme activity in soil organic matter decomposition. *Soil Biol. Biochem.* **71**, 68–75 (2014).

45. S. Buessecker, A. F. Sarno, M. C. Reynolds, R. Chavan, J. Park, M. F. Ortiz, A. G. Pérez-Castillo, G. P. Pisco, J. D. Urquiza-Muñoz, L. P. Reis, J. Ferreira-Ferreira, J. M. Furtunato Maia, K. E. Holbert, C. R. Penton, S. J. Hall, H. Gandhi, I. G. Boëchat, B. Gücker, N. E. Ostrom, H. Cadillo-Quiroz, J. M. F. Maia, K. E. Holbert, C. R. Penton, S. J. Hall, H. Gandhi, I. G. Boëchat, B. Gücker, N. E. Ostrom, H. Cadillo-Quiroz, Coupled abiotic-biotic cycling of nitrous oxide in tropical peatlands. *Nat. Ecol. Evol.*, **6**, 1181–1890 (2022).

46. D. Lewicka-Szczebak, J. Augustin, A. Giesemann, R. Well, Quantifying N_2O reduction to N_2 based on N_2O isotopocules—Validation with independent methods (helium incubation and ^{15}N gas flux method). *Biogeosciences* **14**, 711–732 (2017).

47. F. M. Soper, P. M. Groffman, J. P. Sparks, Denitrification in a subtropical, semi-arid north American savanna: Field measurements and intact soil core incubations. *Biogeochemistry* **128**, 257–266 (2016).

48. J. Shan, R. A. Sanford, J. Chee-Sanford, S. K. Ooi, F. E. Löffler, K. T. Konstantinidis, W. H. Yang, Beyond denitrification: The role of microbial diversity in controlling nitrous oxide reduction and soil nitrous oxide emissions. *Glob. Chang. Biol.* **27**, 2669–2683 (2021).

49. Z. Wang, N. Vishwanathan, S. Kowaliczko, S. Ishii, Clarifying microbial nitrous oxide reduction under aerobic conditions: Tolerant, intolerant, and sensitive. *Microbiol. Spectr.* **11**, e0470922 (2023).

50. S. Leitner, P. M. Homyak, J. C. Blankinship, J. Eberwein, G. D. Jenerette, S. Zechmeister-Boltenstern, J. P. Schimel, Linking NO and N_2O emission pulses with the mobilization of mineral and organic N upon rewetting dry soils. *Soil Biol. Biochem.* **115**, 461–466 (2017).

51. H. Shulman, "Atmospheric nitrogen deposition changes microbial nitrogen cycling in desert soils," thesis, UC Riverside (2022).

52. M. D. Kenichiro Suzuki, E. Collins, K. K. Iijima, Chemotaxonomic characterization of a radiotolerant bacterium, *Arthrobacter radiotolerans*: Description of *Rubrobacter radiotolerans* gen. nov., comb. nov. *FEMS Microbiol. Lett.* **52**, 33–40 (1988).

53. M. Y. Chen, S. H. Wu, G. H. Lin, C. P. Lu, Y. T. Lin, W. C. Chang, S. S. Tsay, *Rubrobacter taiwanensis* sp. nov., a novel thermophilic, radiation-resistant species isolated from hot springs. *Int. J. Syst. Evol. Microbiol.* **54**, 1849–1855 (2004).

54. N. C. Lawrence, C. G. Tenesaca, A. VanLoocke, S. J. Hall, Nitrous oxide emissions from agricultural soils challenge climate sustainability in the US Corn Belt. *Proc. Natl. Acad. Sci. U.S.A.* **118** (2021).

55. J. D. M. Granger, D. M. Sigman, M. G. Prokopenko, M. F. Lehmann, P. D. Tortell, A method for nitrite removal in nitrate N and O isotope analyses. *Limnol. Oceanogr. Methods.* **4**, 205–212 (2006).

56. W. H. Schlesinger, J. F. Reynolds, G. L. Cunningham, L. F. Huenneke, W. M. Jarrell, R. A. Virginia, W. G. Whitford, Biological feedbacks in global desertification. *Science* **247**, 1043–1048 (1990).

57. M. E. Fenn, S. Jovan, F. Yuan, L. Geiser, T. Meixner, B. S. Gimeno, Empirical and simulated critical loads for nitrogen deposition in California mixed conifer forests. *Environ. Pollut.* **155**, 492–511 (2008).

58. J. O. Sickman, A. E. James, M. E. Fenn, A. Bytnerowicz, D. M. Lucero, P. M. Homyak, Quantifying atmospheric N deposition in dryland ecosystems: A test of the Integrated Total Nitrogen Input (ITNI) method. *Sci. Total Environ.* **646**, 1253–1264 (2019).

59. B. B. Osborne, C. M. Roybal, R. Reibold, C. D. Collier, E. Geiger, M. L. Phillips, M. N. Weintraub, S. C. Reed, Biogeochemical and ecosystem properties in three adjacent semiarid grasslands are resistant to nitrogen deposition but sensitive to edaphic variability. *J. Ecol.*, **110** (2022), 1615, 1631.

60. P. M. Homyak, K. T. Vasquez, J. O. Sickman, D. R. Parker, J. P. Schimel, Improving nitrite analysis in soils: Drawbacks of the conventional 2 M KCl extraction. *Soil Sci. Soc. Am. J.* **79**, 1237–1242 (2015).

61. E. A. Davidson, P. M. Vitousek, P. A. Matson, R. Riley, G. García-Méndez, J. M. Maass, Soil emissions of nitric oxide in a seasonally dry tropical forest of México. *J. Geophys. Res.* **96**, 15439 (1991), 15445.

62. H. M. Andrews, A. H. Krichels, "Handr003/TraceGasArray: v1.1 (v1.1)" (Zenodo, 2022); https://zenodo.org/records/7246428.

63. L. Philippot, S. Piutti, F. Martin-Laurent, S. Hallet, J. C. Germon, Molecular analysis of the nitrate-reducing community from unplanted and maize-planted soils. *Appl. Environ. Microbiol.* **68**, 6121–6128 (2002).

64. D. S. Jenkinson, D. S. Powlson, The effects of biocidal treatments on metabolism in soil—I. Fumigation with chloroform. *Soil Biol. Biochem.* **8**, 167–177 (1976).

65. P. M. Homyak, M. Kamiyama, J. O. Sickman, J. P. Schimel, Acidity and organic matter promote abiotic nitric oxide production in drying soils. *Glob. Chang. Biol.* **23**, 1735–1747 (2017).

66. D. Keeling, The concentration and isotopic abundances of atmospheric carbon dioxide in rural areas. *Geochim. Cosmochim. Acta* **13**, 322–334 (1958).

67. D. W. T. Griffith, Calibration of isotopologue-specific optical trace gas analysers: A practical guide. *Atmos. Meas. Tech.* **11**, 6189–6201 (2018).

68. R. C. Team, "R: A language and environment for statistical computing" (R Foundation for Statistical Computing, 2022); www.r-project.org.

69. C. Ranjan, V. Najari, "Package "nlcor": Compute nonlinear correlations" (2020).

70. D. B. Schwede, G. G. Lear, A novel hybrid approach for estimating total deposition in the United States. *Atmos. Environ.* **92**, 207–220 (2014).

71. A. H. Krichels, P. M. Homyak, E. L. Aronson, J. O. Sickman, J. Botthoff, A. C. Greene, H. M. Andrews, H. Shulman, S. Piper, G. D. Jenerette, Soil NH_3 emissions across an aridity, soil pH, and N deposition gradient in southern California. *Elementa* **11**, 00123 (2023).

72. C. Daly, W. P. Gibson, G. H. Taylor, M. K. Doggett, J. I. Smith, Observer bias in daily precipitation measurements at United States cooperative network stations. *Bull. Am. Meteorol. Soc.* **88**, 899–912 (2007).

73. D M Sigman, K L Casciotti, M Andreani, C Barford, M Galanter, J K Böhlke, A bacterial method for the nitrogen isotopic analysis of nitrate in seawater and freshwater. *Anal. Chem.* **73**, 4145–4153 (2001).
